# Exploiting the Oxygen Inhibitory Effect on UV Curing in Microfabrication: A Modified Lithography Technique

**DOI:** 10.1371/journal.pone.0119658

**Published:** 2015-03-06

**Authors:** Jafar Alvankarian, Burhanuddin Yeop Majlis

**Affiliations:** Institute of Microengineering and Nanoelectronics, National University of Malaysia, Bangi, Selangor, Malaysia; Texas A&M University, UNITED STATES

## Abstract

Rapid prototyping (RP) of microfluidic channels in liquid photopolymers using standard lithography (SL) involves multiple deposition steps and curing by ultraviolet (UV) light for the construction of a microstructure layer. In this work, the conflicting effect of oxygen diffusion and UV curing of liquid polyurethane methacrylate (PUMA) is investigated in microfabrication and utilized to reduce the deposition steps and to obtain a monolithic product. The conventional fabrication process is altered to control for the best use of the oxygen presence in polymerization. A novel and modified lithography technique is introduced in which a single step of PUMA coating and two steps of UV exposure are used to create a microchannel. The first exposure is maskless and incorporates oxygen diffusion into PUMA for inhibition of the polymerization of a thin layer from the top surface while the UV rays penetrate the photopolymer. The second exposure is for transferring the patterns of the microfluidic channels from the contact photomask onto the uncured material. The UV curing of PUMA as the main substrate in the presence of oxygen is characterized analytically and experimentally. A few typical elastomeric microstructures are manufactured. It is demonstrated that the obtained heights of the fabricated structures in PUMA are associated with the oxygen concentration and the UV dose. The proposed technique is promising for the RP of molds and microfluidic channels in terms of shorter processing time, fewer fabrication steps and creation of microstructure layers with higher integrity.

## Introduction

Fabrication of microfluidic devices using elastomeric materials such as thermal-curing polydimethylsiloxane (PDMS) by soft lithography/molding is a common research approach[1]. Another fabrication method is direct ultraviolet (UV) which is applicable in UV-curable materials such as polyurethane methacrylate (PUMA) and involves the following process steps: resin deposition, UV-exposure, and post-exposure development. The casting technique provides better structural integrity than UV lithography because in the lithography of a new multilayer device, multiple resin depositions must be performed with interlayer bonding, which creates a structure that is not as strong as a single molded component[2].

In photolithography, as UV rays travel into the resin the cross-linking of pre-polymer is started and the front edge of curing advances parallel to the photomask into the lower layers of the photopolymer and develops the curing initialization zone. Cabral et al. have fully investigated the concept of frontal photopolymerization (FPP) in the absence of oxygen inhibition for application in the RP of a multilevel micromixer[3]. The composition of a typical UV-curable material contains a photo-initiator for absorbing the UV light and generating the radical species for curing initialization. The produced radicals stop the polymerization process after termination of exposure to obtain a sharp image. The polymerization rate is inhibited by air because the oxygen molecules scavenge the radical species needed for crosslinking initialization. Diffusion of oxygen from a high concentration zone into a pre-polymer resin during UV curing requires an additional amount of photo-initiator and UV energy to consume the dissolved and diffused oxygen [4–9]. Dendukuri et al. have reported a general modeling of the radical UV curing under the inhibitory effect of oxygen and the resulting loss of optical transparency [10].

Using a photomask with proximity contact produces a highly tacky surface that has incompletely curing and is unsuitable for interlayer reversible covalent bonding in microfluidic devices. To overcome the negative role of oxygen in polymerization different techniques have been proposed to reduce the concentration of oxygen surrounding the photopolymer. The hard contact of the photomask or an impermeable transparency placed over the resin surface can allow the UV rays to pass with least attenuation while also having the effect of almost stopping ambient oxygen penetration into the exposed top surface of the resin. Haraldsson et al. employed the technique of the contact photomask for pattern transfer in contact liquid photolithographic polymerization (CLiPP) in the fabrication of three-dimensional microfluidic devices using a typical liquid polymer precursor composition [11]. Similarly, Kuo et al. and the authors of the present work similarly have employed an impermeable transparency cover sheet for complete curing of hard-PUMA [12] and soft-PUMA [13] in UV molding. A cover sheet of gas-permeable material such as PDMS or the vacuuming of the air covering the sample can reduce the oxygen penetration rate and leave a surface with less tackiness but it is still incompletely cured, as reported by Jeong et al. [14, 15]. Another method has been reported by Studer et al. in which the thin film surface is exposed to a composition of air and carbon dioxide to overcome the incomplete curing of a photopolymer coating due to oxygen inhibition [16, 17].

Despite such efforts to reduce the negative effects of oxygen, researchers have also reported methods to exploit the oxygen inhibition of UV curing in microfabrication. The bonding of the layers of a microfluidic channel with semi-cured surfaces of a UV-curable material has been characterized under different conditions of oxygen diffusion and UV-exposure time[15]. A multiscale hierarchical structure has been produced by Jeong et al. [14] using capillary force lithography (CFL) in a two-step process involving sequential micro- and nano-molding and by exploiting the partially cured surface of UV-sensitive material. The modeling and fabrication of cuboidal microparticles by using a combination of FPP and the inhibitory effect of oxygen in the UV curing of resin and by applying CFL in an all-PDMS microfluidic channel has been reported by Dendukuri et al. [10].

In this article, the inhibitory effect of the diffusion of oxygen on the UV curing of PUMA (Polydiam, UK) is utilized in a modification of SL for microfabrication. The use of PUMA as the main substrate has been characterized previously for microfluidic applications by the authors of the present work [13, 18, 19]. Use of the new technique proposed in this article results is one less fabrication step, shorter process time and higher integrity of the microstructure layer, giving the modified lithography process some distinct advantage over SL. In the modified process, maskless exposure lets the oxygen diffuse into the resin and leaves a partially cured film on top for microstructure patterning while the base layer is constructed. Several microstructures are fabricated using the introduced technique. The UV-exposure dose is used to control the oxygen inhibition of the top surface of the resin and the conversion rate to solid PUMA. The experimental results are explained by a one-dimensional equation of oxygen diffusion, UV absorption of PUMA, and the rate of UV curing of resin.

## Materials and Methods

### Incorporating oxygen diffusion and UV curing in SL

**Fig 1:**
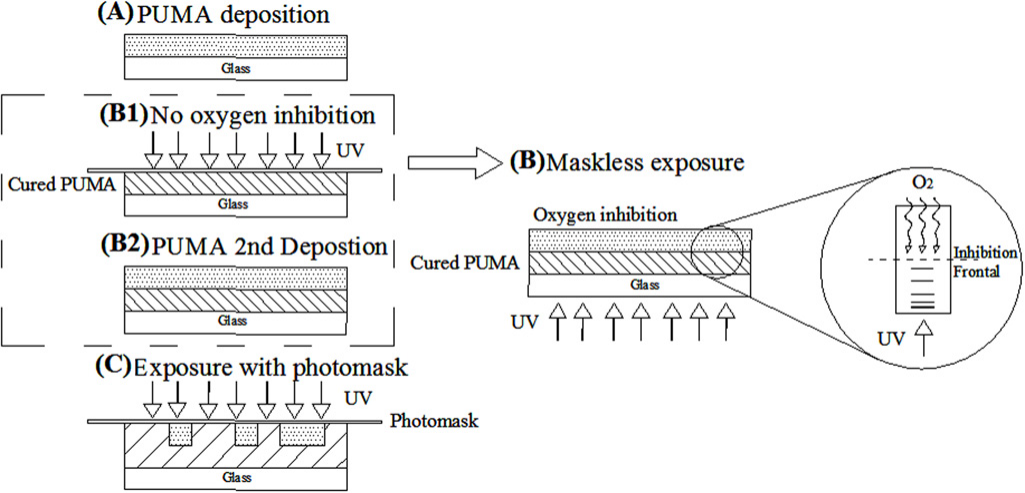
Standard lithography and modified steps. **(A)** Resin deposition. **(B1)** Full curing of base layer. **(B2)** Resin deposition for microstructure patterning. **(B3)** Replacement for step B1 and B2. **(C)** Exposure with photomask for pattern transfer.

The general fabrication of a microstructure in PUMA resin is performed by SL in the following steps, as illustrated in Fig. 1: A) resin deposition for the base layer, B1) complete UV curing of the base layer, B2) resin deposition for the structure layer, and C) microstructure pattern transfer onto uncured polymer resin followed by a final step of washing the un-crosslinked PUMA. Taking into consideration that oxygen diffuses into open air from the top surface of the resin and also frontal movement of UV polymerization in the direction of the photo rays, step (B) is designed to substitute steps (B1) and (B2) in SL. The mobility of molecules of oxygen in resin is considerably higher than in solid polymer[9], therefore the frontals of oxygen inhibition and resin cross-linking merge into the borderline separating the deposited film in two sections of cured and uncured PUMA. The position of the frontal border can be controlled by adjusting UV power and the available atmospheric air. At low UV power, full crosslinking takes longer, therefore the inhibition frontal is deeper in the polymer resin[10]. Polymer resins with higher viscosity have a low level of oxygen inhibition [9].

### Modeling oxygen inhibition frontal

The crosslinking of PUMA resin starts by absorbing part of the radiated UV light and producing the radicals needed in polymerization while the reaction is inhibited by oxygen diffused in the pre-polymer. The molecules of oxygen are consumed via scavenging the radicals and terminating the reaction. The rate of change of UV intensity, I(z), is expressed by Beer-Lambert’s law [10] given by:

$$\frac{\partial I\left(z\right)}{\partial z}=-\mu I\left(z\right)$$ (1)

where the attenuation coefficient, μ (mm^-1^), depends on the chemistry of the material and is determined experimentally. Assuming that μ is constant throughout the resin thickness before polymerization, integration of equation (1) gives:

$$I\left(z\right)=I_{0}e^{-\mu z}$$ (2)

where I_0_ is the light intensity at the first incidence point in the photopolymer. The time-dependent and one-dimensional equation of oxygen concentration in the pre-polymer,C_*o*2_, is determined by Fick’s diffusion model given by:

$$\frac{\partial C_{o_{2}}}{\partial t}=D_{o_{2}-re\sin}\frac{\partial^{2}C_{o_{2}}}{\partial z^{2}}-kC_{o_{2}}$$ (3)

Where *D*_*o*2-*resin*_ is the diffusion coefficient and is also determined experimentally. The oxygen consumption rate, k, is proportional to the concentration of radical species, which is also related to local light intensity. In the first-order reactions of polymerization [20], the coefficient of consumption of oxygen, k, can be considered a function of local UV absorbance of the material which is given by:

$$k\propto \mu I_{0}e^{-\mu z}$$ (4)

Conversion of the monomers to polymer as a function of time and depth is defined by the parameter *ϕ*(*z*,*t*), which has a range between 0.0 and 1.0. The rate of change of this variable is formulated as a function of the multiplication of key parameters of UV intensity, oxygen inhibition, and the available material for conversion and is described by:

$$\frac{\partial \varphi \left(z,t\right)}{\partial t}=K\left(1-\theta \right)\left(1-\varphi \left(z,t\right)\right)I\left(z\right)$$ (5)

where $\theta =\frac{C_{o_{2}}}{C_{o_{2}-surface}}$is the dimensionless oxygen concentration, and *K* is the resin conversion rate coefficient and is determined experimentally [3]. The resin is said to be polymerized when the conversion fraction has increased from 0.0 to a critical minimum value of *ϕ*_*c*_ and the resin becomes solid structurally and cannot be washed away [3]. Further exposure of the resin will increase the convection fraction up to the maximum of 1.0 while improving the hardness of the material.

By knowing the initial oxygen concentration in the resin, *C*_*o*2-*z*_(*t*_0_) = *C*_*o*2-*surface*_, and the boundary conditions on the two sides of the PUMA film, at z = L, *C*_*o*2 =_ *C*_*o*2-*surface*_ and at z = 0,$\frac{\partial C_{o_{2}}}{\partial z}=0$, the above equations are solved to determine the level of oxygen concentration and conversion fraction as a function of time and z during UV curing.

If the diffusion of the oxygen from the top surface is stopped, then we have $\frac{\partial C_{o_{2}}}{\partial z}=0$ throughout the resin PUMA thickness and Fick’s diffusion model is simplified to:

$$\frac{\partial C_{o_{2}}}{\partial t}=-kC_{o_{2}}$$ (6)

Then, the closed form solution of equations 5 and 6 are obtained as:

$$\theta =e^{-kt}$$ (7)

$$\varphi =1-e^{-KI(z)(t+\theta /k-1/k)}$$ (8)

If the diffusion and consumption of oxygen inside the resin reaches an equilibrium condition, then we have a steady state, where $\frac{\partial C_{o_{2}}}{\partial t}=0$ throughout the uncured resin PUMA thickness and the diffusion model is simplified to:

$$D_{o_{2}-re\sin}\frac{\partial^{2}C_{o_{2}}}{\partial z^{2}}-kC_{o_{2}}=0$$ (9)

The closed form solution of equation 9 is given by:

θ=coshmz−tanhmL×sinhmz (10)

$$m=\sqrt{\frac{k}{D_{o_{2}-re\sin}}}$$ (11)

where L is the thickness of the uncured resin. For a conversion ratio to reach the amount of *ϕ*_*c*_, it is necessary that by consumption of oxygen, *θ* is reduced to less than a critical value of *θ*_*c*_ [15].

### Characterization of UV curing

Several tests were designed to evaluate the optical transparency, oxygen inhibitory effect, and polymerization rate and depth in PUMA resin during the UV-curing process.

The UV transmission of the resin is measured by examining samples with different thicknesses of 1.0–10.0 mm prepared using a mold and exposed to UV light with a wavelength of 365 nm. The exposure doses received by the samples at different time steps are calculated by multiplying of the incident dose and transmission coefficient of the resin. The attenuation coefficient is defined according to equation 1, which represents the amount of light absorbed for polymerization of the resin.

**Fig 2:**
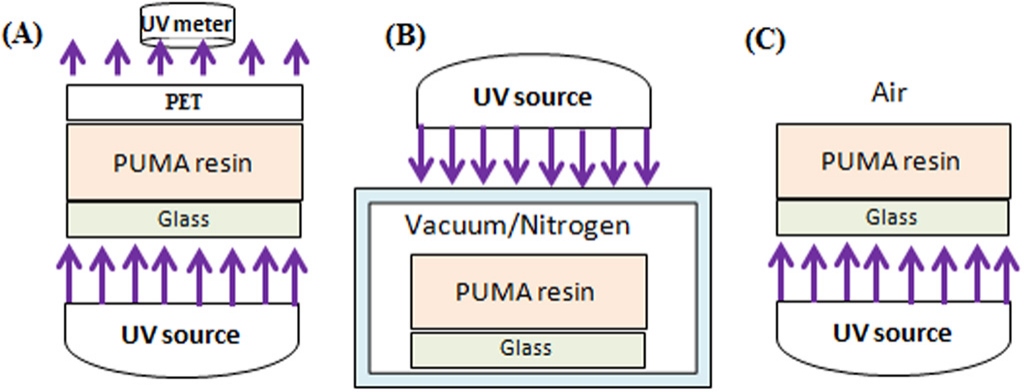
Curing processes of PUMA resin. **(A)** Full curing by covering the resin with transparency sheet. **(B)** Control of curing by applying low vacuum or nitrogen shower. **(C)** Partial curing by exposing top surface of the resin to air.

For depth of cure measurements, PUMA samples with a thickness of 4.0 mm are prepared and covered with a sheet of transparent and low-oxygen permeability polyethylene terephthalate (PET). The samples are exposed to UV radiation at different doses from the bottom surface and at each time step after washing the uncured resin in an ultrasonic agitator, the thickness of polymerized material is measured. The PUMA film is formed on a glass support substrate as shown in Fig. 2 (A).

Tests are also conducted on the effects of the vacuum condition and nitrogen gas on full curing of the resin while its top surface is uncovered. As shown in Fig. 2(B), oxygen concentration around the sample is reduced by applying the vacuum condition or covering the PUMA resin by nitrogen shower in a small chamber during UV curing.

Another set of tests is performed to indirectly measure the depth of oxygen diffusion into the PUMA resin. Samples of 1.0 mm thickness prepared for this experiment are exposed to a range of UV doses from the bottom surface while the top surface is left without a photomask as depicted in Fig. 2(C). The thickness of the polymerized material is measured at different time steps.

### Setup and preparation

As the UV light source required for the experiments a low-power portable unit (Model UVL-56, Black-Ray, 6 Watts Lamp, 365 nm wavelengths, UVP, Uplard, CA USA) was used in which light is collimated by a parabolic light reflector. The maximum light intensity radiated at a distance of 15.0 cm from the lamp is 518.0 μW/cm^2^ measured by a UV intensity meter. The photomask was created in CAD software and printed on transparency flexible paper using a high-resolution laser printer or by directly printing on a chrome-coated glass.

The samples were cured at low-power radiation so that the curing rate was slowed down and the exposure duration was increased to obtain a wide range of measuring points and better control over the curing process. The characterization procedure was carried out for time duration ranging from 1–30 minutes with steps of 1 minute, covering a UV dose of 31.0–930.0 mJ/cm^2^. After removing the photomask from the supporting substrate it was placed for 20 minutes in an ultrasonic agitator filled partially with water to wash away the uncured resin. The thicknesses of the samples were measured by using an optical microscope (STM6, Olympus, Japan) equipped with a three-axis measurement unit.

### Microfabrication

As a proof of concept of application of the proposed technique to produce microstructures for microfluidic devices, molds of soft lithography, and microstamps, some typical microchannels with positive and negative polarity are fabricated. First, a substrate of PUMA resin with a thickness of ≈1.0 mm, which is uncovered, is exposed to UV radiation from the bottom surface. Then, the photomask with patterns of microchannels is placed on top surface and UV curing is applied to the top surface. The first UV-exposure dose determines the thickness of the base layer and the height of the microstructure, then the second dose transfers the patterns of the microchannels onto the uncured resin. After that, the photomask is removed and the un-crosslinked material is washed away by agitation in deionized water for 20 minutes followed by drying by nitrogen gas. The patterned polymer is then post-cured for an additional 5 minutes. The overall process takes about 30 minutes.

## Results and Discussion

### Analysis and measurements

**Fig 3:**
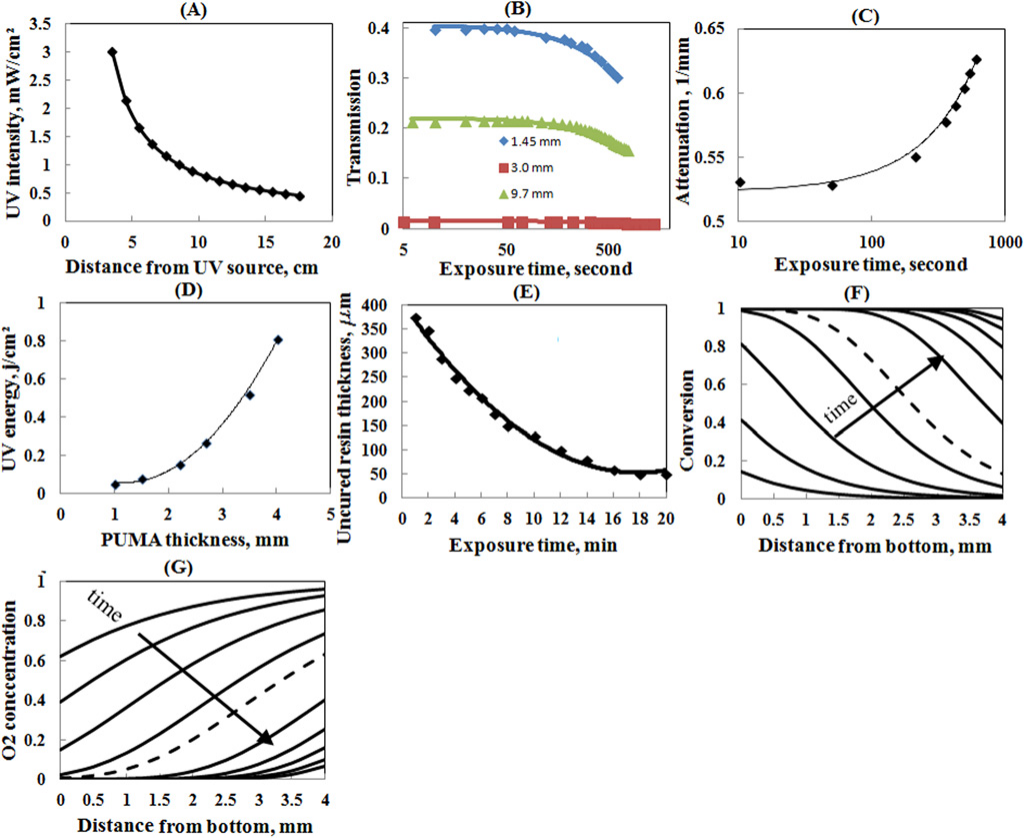
(A) Irradiation dose at distances from the source. (B) UV transmission of PUMA of different thicknesses for different exposure duration and lamp-to-sample distance of 13.0cm. (C) Attenuation of PUMA resin per millimeter of film thickness for different exposure durations and lamp-to-sample distance of 13.0 cm. (D) UV dose required for complete cure of PUMA resin of different thicknesses. (E) Thickness of PUMA resin remaining uncured for PUMA film of 1.0mm thickness exposed for different curing durations at lamp-to-sample distance of 3.5cm. (F) and (G) Conversion ratio and dimensionless concentration of oxygen versus depth for exposure duration of 5 to 360 seconds for covered sample. Dashed line corresponds to 60 seconds.

The graphs in Fig. 3 summarize the results of the characterization tests performed to find the frontal of the polymerization and oxygen inhibition zone of the PUMA resin during the curing process.

Measurement of the UV radiation intensity was performed using a UV radiometer positioned at different distances from the source. Fig. 3(A) shows that the UV intensity of the utilized radiation source is reduced inversely with distance from ~3.0 to ~0.5 mWcm^-2^ when the sample is repositioned in the range of 3.5–17 cm from the light source. The graph in Fig. 3(B) shows the results of the data measurement of optical transmission versus exposure time. Initially, when most of the PUMA film is in a liquid state, the transmission at different thicknesses of material (1.34mm, 3.0mm, and 9.7mm) is almost constant, followed by slight decline at higher exposure doses when the resin has been mostly polymerized. By using the obtained results for transmission rate and applying Beer-Lambert’s law in equation 2, the UV attenuation coefficient for PUMA can be calculated. The graph in Fig. 3(C) shows an increase of μ from ~0.53 to 0.63 when the exposure time increases from 10 to 600 seconds for a sample-to-UV-source distance of 13.0 cm. The UV light has darkening effect on PUMA during photo-polymerization. This phenomenon shows itself by an increase of μ by exposure time.

As shown in the graph in Fig. 3(D), while the top surface of the resin is covered with a sheet of PET, the UV dose required for complete curing of PUMA with thicknesses of 1.0–4.0 mm, increases nonlinearly from 50.0 to 810.0 Jcm^-1^ corresponding to exposure times of 24–380 seconds and sample-to-UV-source distance of 4.0 cm. The condition of complete curing can be defined when the conversion rate of resin to polymer has reached to ~100.0%. Physically, this condition is met when the mechanical properties of hardness and modulus of elasticity reach their maximum values after UV curing. The experiment was repeated for samples with an uncovered top surface in an ambient and also a vacuum pressure condition of ~1.0bar. Due to the inhibitory effect of oxygen, a thin layer of resin on the top surface of the film remained uncured under the conditions of the performed tests.

Using equations 7 and 8, the conversion rate and oxygen concentration at depths of 0.0–4.0 mm are graphed at increasing curing time of 5, 10, 20, 40, 60, 120, 180, 240, 300, and 360 seconds as shown in Fig. 3(F) and 3(G). For simplicity of the analysis, μ is considered constant for all ranges of incident intensity and exposure time in determining the frontal of polymerization. The reduction of oxygen concentration by consumption with no diffusion from the outside resulted in the rapid conversion of the resin to polymer. As can be seen, at an exposure time of 60 second, *θ* has reduced to less than 10% while the conversion fraction has increased to above 90%.

The graph in Fig. 3(E) shows the experimental results of the thickness of uncured resin of PUMA film versus radiation time when the sample is exposed from bottom surface while its top surface is in direct contact with air. The calculation is based on the initial thickness of the resin film and the thickness of the crosslinked PUMA after completing exposure and washing the uncured resin. For a film thickness of 1.0 mm and exposure time of 1–20 minutes, the thickness of uncured resin is reduced from ~380.0 μm to ~50.0 μm. At a longer exposure time, the rate of consumption of oxygen and diffusion into the liquid resin reaches equilibrium and therefore polymerization is stopped. This effect is noticeable after 18 minutes of exposure when the thickness of the liquid resin is ~50 μm. By covering the substrate with nitrogen, full curing of the PUMA resin was achieved for less than 60 seconds exposure to UV radiation at the same intensity.

### Microchannels for microfluidics

**Fig 4:**
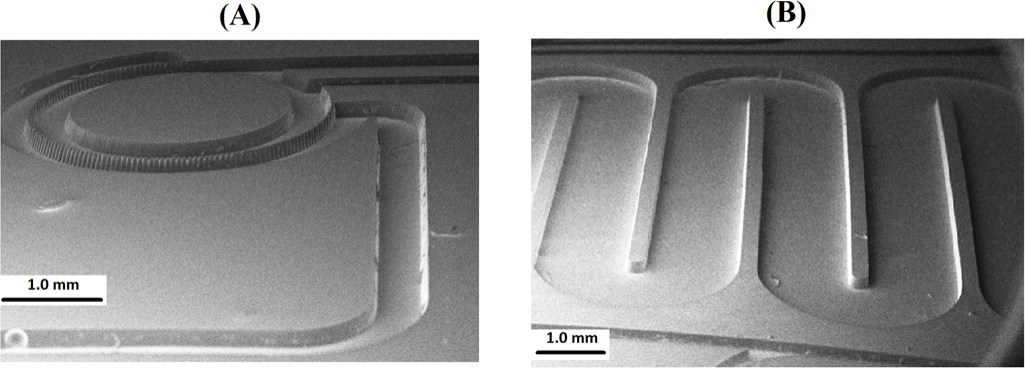
SEM images of a typical microstructure for application in microfluidic devices fabricated using proposed technique. Depth of microstructures in **(A)** and **(B)** is ~200.0 μm and camera angle is 70^°^.

The main application of polymer microchannels is in microfluidic devices. Fig. 4 shows the microscopic images of the microstructures fabricated for this work. To construct a microstructure with a depth of ~200 μm, the first and second exposure was chosen based on the graphs in Fig. 3(D) and 3(E). The fabricated part is comparable to a component made by using the molding process in terms of integrity of the top to the base layer. The limitation of the proposed technique is that the height of the microchannel falls in the range controlled by oxygen diffusion into resin during the UV curing process. The difficulty of fabricating microchannels with high depth but narrow width (aspect ratio > 2.0) arises because it is hard to remove sticky semi-cured or uncured resin without collapsing such microstructures during the long time they undergo ultrasonic agitation in water.

### Fabrication of array of micropillars

**Fig 5:**
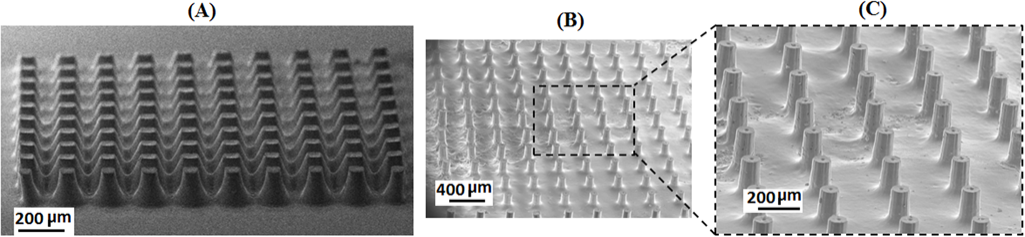
SEM images of an array of micropillars. **(A)** Array of pillars with square cross-section. **(B)** and **(C)** Array of pillars with circular cross-section. Average dimension of each single pillar is 100 μ × 200 μ, (D × H) and aspect ratio ~2.0.

Finding an effective technique is crucial for the microfabrication of arrays of micropillars with different sizes and spacings. As structure with high aspect ratio micropillars are considered a benchmark for demonstrating the performance of a material and a technique in the fabrication of tall structures. There are two critical parameters in pillar array fabrication. First, the pillar aspect ratio (ratio of height to diameter of each single post) is important in microfabrication especially in elastomeric substrates where the aspect ratios exceed certain amounts and the pillar is collapsed or bent to one side. Second, the small spacing between the neighboring posts can make the removal of un-crosslinked pre-polymers difficult leaving arrays of pillars semi-developed (see Fig. 5(A)).

In this work, arrays of pillars with circular cross-section were produced by selecting the proper first exposure dose for defining the root plane. Fig. 5(B) shows a scanning electron microscopy (SEM) image of an array of pillars fabricated at a height set at 200.0 μm by the proper selection of the first exposure time. Each single pillar is ~100.0 μm in diameter and there is 300.0 μm of inter-pillar spacing. The average height of the pillars is ~200.0 μm. Calculation of the average aspect ratio gives a value of ~2.0 (height of pillar divided by its diameter) in a well-formed array of vertically standing pillars. Fig. 5(C) shows that there is a root broadening effect on each single pillar. This effect originates from over-curing of the resin during the second exposure which makes the cleaning process of the semi-cured material difficult. Ultrasonic agitation in water was able to remove the unexposed resin in the inter-pillar area within 20 minutes giving a clean surface without damaging the pillars crosslinked to the root plane. The sample was sputter coated with ~200A° gold before SEM. Integration of the structure layer with the base layer was through a good natural bonding between two layers of the same material.

### Microstructure with negative polarity

**Fig 6:**
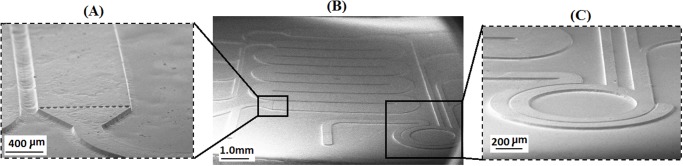
SEM images of a negative polarity microstructure fabricated by two-step exposure lithography process using PUMA resin. **(B)** Overall view of structure. **(A)** and **(C)** Close-up images of microstructures. Height of structure is ~50.0 μm and camera angle is 70^°^.

PUMA as a material with elastomeric properties (modulus of elasticity ≈1.35 MPa) and low hardness (Shore A45) has good potential to be used as the main substrate of a mold used repeatedly in soft lithography or micro-printing technologies. Fig. 6 shows the SEM images of microstructures with negative polarity. The height of the structure layer was adjusted to ~50.0 μm by using the proposed technique of microfabrication. The uniformity of the structure height in the area of 2.0 × 2.0 cm was maintained in ±5.0% as measured by the Olympus optical microscope equipped with a coordination measurement system. As shown in Fig. 6(A), after removing the photomask the upper surface of the microstructure is quite uniform and smooth with sharp edges, which are necessary for good bonding of microchannels. It is noticeable that the structure contours are well formed at the corners. Also, as can be seen in insets of 6(A) and Fig. 6(C) the corners of the walls have a round curvature shape due to difficulty in removing semi-cured resin by ultrasonic agitation when maskless exposure time is longer.

## Conclusion

A novel process for the fabrication of microstructures for applications such as microchannels of microfluidics devices was reported herein. The two-step exposure lithography technique for prototyping applied in this work proved very simple, effective, and was based on the conventional photolithography process and on equipment commonly found in the MEMS labs. According to the SEM images taken, the fabricated microstructures have good quality for use in intended applications. The whole process shown takes a very short time to fabricate a newly designed microfluidic structural layer. Theoretical analysis and the performed experiments demonstrated the reproducibility of the proposed technique for the fabrication of microstructures. While the technique offers the advantages of reducing the number of fabrication steps, increasing the integrity of the microstructure, and reducing the process time, its major drawback lies in the difficulty of removing semi-cured resin from the corners of the microchannels with a high aspect ratio to deliver a high-resolution microstructure. In the described conditions over-washing the microstructure to remove this resin can lead to the collapse of the microstructure.

In summary, the introduced technique has good potential for RP of the new microfluidic devices especially for research works. However, more characterization and optimization must be performed on PUMA or alternative UV-curable materials to improve the technique for the fabrication of microstructures in a wider range of sizes and forms. Materials that are curable solely by the UV radiation are suitable for use in reducing the design-to-device time and in testing new ideas for microfabrication technologies.
